# Ferroptosis-Inducing Nanomedicine for Cancer Therapy

**DOI:** 10.3389/fphar.2021.735965

**Published:** 2021-12-20

**Authors:** Yang Wang, Tianfu Liu, Xiang Li, Hui Sheng, Xiaowen Ma, Liang Hao

**Affiliations:** ^1^ Department of Chemistry, School of Forensic Medicine, China Medical University, Shenyang, China; ^2^ Key Laboratory of Forensic Bio-evidence Sciences, Shenyang, China; ^3^ China Medical University Center of Forensic Investigation, Shenyang, China; ^4^ China Medical University-The Queen’s University of Belfast Joint College, China Medical University, Shenyang, China; ^5^ First Department of Clinical Medicine, China Medical University, Shenyang, China; ^6^ Physical College, Liaoning University, Shenyang, China; ^7^ Second Department of Clinical Medicine, China Medical University, Shenyang, China; ^8^ Department of Biochemistry and Molecular Biology, China Medical University, Shenyang, China

**Keywords:** ferroptosis, nanomedicine, cancer therapy, combination strategies, ROS

## Abstract

Ferroptosis, a new iron- and reactive oxygen species–dependent form of regulated cell death, has attracted much attention in the therapy of various types of tumors. With the development of nanomaterials, more and more evidence shows the potential of ferroptosis combined with nanomaterials for cancer therapy. Recently, there has been much effort to develop ferroptosis-inducing nanomedicine, specially combined with the conventional or emerging therapy. Therefore, it is necessary to outline the previous work on ferroptosis-inducing nanomedicine and clarify directions for improvement and application to cancer therapy in the future. In this review, we will comprehensively focus on the strategies of cancer therapy based on ferroptosis-inducing nanomedicine currently, elaborate on the design ideas of synthesis, analyze the advantages and limitations, and finally look forward to the future perspective on the emerging field.

## 1 Introduction

Cancer is a public health problem worldwide, which cannot be ignored currently, with the incidence gradually increasing year by year. As one of the deadliest diseases in the world, its prevalence has been more than 10 million mortalities annually (Siegel et al., 2021). Current cancer conventional treatments include surgery, radiotherapy, and chemotherapy which have various shortcomings affecting the effective treatment (Johnstone et al., 2002; Esposito et al., 2019; Poon et al., 2021). With the deep understanding of precision medicine recently, targeted therapy and immunotherapy have great progress in cancer treatment (Pham et al., 2018; Adams et al., 2019; Chen et al., 2020a; Rana and Bhatnagar, 2021). However, due to off-target effect, potential toxicity, and drug delivery barriers, these emerging treatment methods fell short of expectation and do not have wide clinical applications.

Ferroptosis, as a form of iron-dependent regulatory cell death, has played an important role in tumor suppression and treatment (Xu et al., 2021a). Targeting ferroptosis has become a promising tumor treatment strategy. In 2001, a unique regulated cell death form by the oxidative stress of nerve cells had been found (Tan et al., 2001). It was first put forward to the conception of ferroptosis by Stockwell in 2012 (Dixon et al., 2012). Different from existing forms of cell death such as apoptosis, autophagy, necrosis, and pyrolysis, the process of ferroptosis mainly includes the iron-dependent Fenton reaction and lipid peroxidation–producing ROS (reactive oxygen species) (Lu et al., 2018; Hassannia et al., 2019; Mou et al., 2019). On the one hand, the iron (III) in the ferritin complex can enter the cell through the glycoprotein transferrin and its carrier protein transferrin receptor (Xu et al., 2021b). Imported iron is reduced and dissociated from the complex to form iron (II). Increased iron promotes the Fenton reaction between iron and hydrogen peroxide (H_2_O_2_), which in turn generates ROS (Liu et al., 2021a; Vitalakumar et al., 2021; Xie and Guo, 2021). On the other hand, the polyunsaturated fatty acid (PUFA-LPs-OH) in the cell membrane can be oxidized by ROS and lipoxygenase to form lipid peroxidation (PUFA-LPs-OOH), which generates toxic lipid-free radicals resulting in cell death (Xie et al., 2016). The toxic PUFA-LPs-OOH can be reduced to nontoxic PUFA-LPs-OH by glutathione peroxidase 4 (GPX4). On the contrary, glutathione (GSH) can be oxidized to oxidized GSH (GSSG). Therefore, GPX4 is the key regulator for ferroptosis (Seiler et al., 2008; Seibt et al., 2019; Song et al., 2020). The synthesis of GSH in the cell requires the cystine (Cys)/glutamic acid (Glu) transport system with different directions to transfer Cys into the cell. In this process, system Xc-, including SLC7A11 and SLC3A2, plays an important role (Hong et al., 2021; Liang and Sun, 2021; Ma et al., 2021; Ortiz-Rodriguez et al., 2021). Therefore, according to the mechanism of ferroptosis mentioned above, regulation of the Fenton reaction and the inhibiting activity of the GPX4 enzyme are the two most effective strategies for inducing ferroptosis in tumor cells.

With the regulation mechanisms and signaling pathways of ferroptosis clarified, ferroptosis has attracted increasing attention in cancer therapy over the years (Xu et al., 2019; Nguyen et al., 2020; Xu et al., 2021c). Some small molecule ferroptosis inducers have the potential to become drugs on the treatment of cancer (Xu et al., 2021b). These drugs mainly include system Xc-inhibitors (erastins, sulfasalazine, and sorafenib) and GPX4 inhibitors (RSL3 and altretamine), inducing GPX4 degradation (FIN56) and GSH depletion (buthionine sulfoximine and DPI2) (Lachaier et al., 2014; Yang et al., 2014; Woo et al., 2015; Mai et al., 2017; Gaschler et al., 2018; Wang et al., 2019; Wang et al., 2021a; Cheng et al., 2021; Zhuang et al., 2021). Although some of them have been clinically approved, most of them are in the research stage due to poor solubility, non-specific distribution, and unpredictable side effects. Notably, the rapid development of nanotechnology provides more possibilities for the application of ferroptosis in tumor treatment (Asghari et al., 2019; He et al., 2019; Matos et al., 2019). Because of its unique structure and properties, as carriers, nanomaterials are not only made up for the limitation of traditional drugs but also have introduced new specific features to produce synergy with small molecule drugs, such as generation of ROS or depletion of GSH. Moreover, the nanomaterials even can be the responder as energy synergy therapy.

Due to the potential of ferroptosis in cancer therapy and advantages of nanotechnology in application in the medical field, there are many opportunities and challenges for combination between target ferroptosis and nanotechnology for cancer therapy (Liang et al., 2019). Therefore, it is necessary to summarize the latest work and progress in ferroptosis-inducing nanomedicine for cancer therapy. Meanwhile, the diversity of the biological system and the complexity of clinical application give both challenges and opportunities for further development of ferroptosis-inducing nanomedicine for cancer therapy. Therefore, it is timely to elaborate on the latest advances in this field. According to the mechanism of ferroptosis, from accelerating the Fenton reaction, inhibiting the activity of GPX4, exogenous delivery of lipid peroxides, and combination with conventional therapy, this progress report focused on recent advances of the construction of ferroptosis-inducing nanomedicine and application in cancer therapy.

## 2 Emerging Nanomedicine-Inducing Ferroptosis for Cancer Therapy

### 2.1 Inducing Ferroptosis by Accelerating the Fenton Reaction

The Fenton reaction was first described by H. J. H. Fenton in 1894 (Hirschhorn and Stockwell, 2019), whose reaction equation is described as follows (Li et al., 2021a; Wang et al., 2021b) (Yang et al., 2021a):

$$\begin{array}{l} F{\text{e}}^{2+}+H_{2}O_{2}=Fe^{3+}+\cdot OH+HO^{-}\\ Fe^{3+}+H_{2}O_{2}=F{\text{e}}^{2+}+\cdot OOH+H^{+} \end{array}$$

In tumor cells, H_2_O_2_ released by the mitochondria react with endogenous Fe^2+/3+^ to generate highly toxic hydroxyl radicals and result in tumor cell death *via* inducing ferroptosis. According to the mechanism of the Fenton reaction, various ferroptosis-inducing nanomedicines for cancer therapy have been developed (Qian et al., 2019). Among them, the design of high-performance nanocatalysts or directly increasing the concentration of reactants to accelerate the Fenton reaction is the most effective strategy.

#### 2.1.1 Accelerating the Fenton Reaction by High-Performance Nanocatalysts

The Fenton reaction is the most direct way to induce ferroptosis. As the important factor to accelerate the reaction, several nanocatalysts based on the Fenton reaction have been developed for cancer treatment (Meng et al., 2020; Wang et al., 2020; Wang et al., 2021c; Kim et al., 2021).

Furthermore, Shi et al*.* synthesized a new type of single-atom Fe nanocatalyst, in which Fe atoms are isolated in nitrogen-doped carbon, and PEGylation of the outer layer of the catalyst can enhance structural stability and effectively nanocatalyze the Fenton reaction for tumor treatment (Huo et al., 2019). The single-atom Fe catalyst with high catalytic performance would be activated by the weak acidic microenvironment of the tumor and effectively induce the Fenton reaction in the tumor site, which would generate a large amount of toxic hydroxyl radicals (Tian et al., 2021). According to the mechanism of the Fenton reaction, on the one hand, radicals catalyzed by the single-atom Fe nanocatalyst can induce cell apoptosis, and the accumulation of lipid peroxides can lead to ferroptosis of tumor cells as well. The synergistic effect of the catalyst has an impressive tumor suppression outcome. Meanwhile, its good biodegradability and biocompatibility show the potential for application *in vivo*.

**FIGURE 1:**
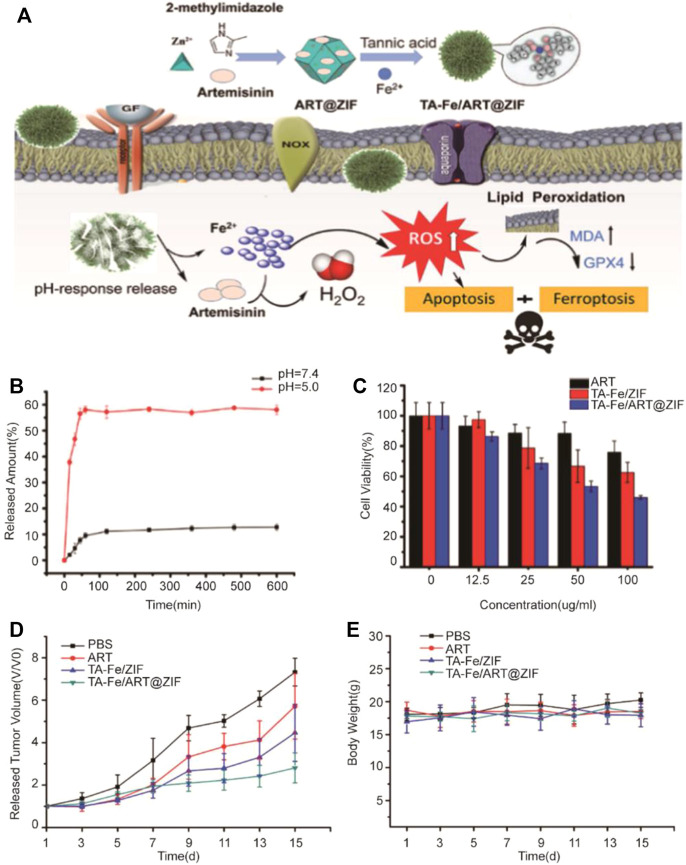
**(A)** Illustration of preparation of nanoparticles and the application in tumor cells; **(B)** Releasing curve of ART from TA-Fe/ART@ZIF nanoparticles *in vitro* at pH 7.4 and 5.0; **(C)** Cytotoxicity of ART, TA-Fe/ZIF, and TA-Fe/ART@ZIF; **(D)** Relative tumor volume after treated with PBS, ART, TA-Fe/ZIF, and TA-Fe/ART@ZIF nanoparticles; **(E)** Relative mouse body weight of various groups. (Adapted from Ref. 54 with permission. Copyright © 2021 Nanoscale Research Letters) ART: artemisinin; TA: tannic acid; ZIF: zeolitic imidazolate framework.

Li et al. (2021b) developed tannic acid (TA) and Fe^2+^ coated on zeolite imidazole ester skeleton-8 (ZIF-8) self-assembly, which was used as the carrier to encapsulate artemisinin (ART) (Figure 1). In nanomedicine, ART would catalyze the degradation of ferritin *via* independent autophagy-lysosome pathways, which could increase the amounts of Fe^2+^ in cells and induce ferroptosis (Chen et al., 2020b). As a carrier, ZIF-8 not only had good biocompatibility but also pH-responsive ability. Drug release experiments showed that ART was only released in pH = 5.0 after 10 h to indicate its tumor-targeted release performance. Meanwhile, increased ROS in the cell, accompanied with decreasing GSH and GPX4, can induce markedly enhanced ferroptosis. The nanomedicine had been demonstrated a better ability of human breast cancer model suppression *in vitro* and *in vivo*. The pH-responsive ability of ZIF-8 has already increased the efficacy of artemisinin; if the targets are being introduced into the ZIF-8 shell, it is hopeful to achieve clinical application.

#### 2.1.2 Accelerating the Fenton Reaction by Increasing Concentration of Reactants

Shi et al*.* synthesized amorphous iron nanoparticles (AFeNPs) with suitable particle size and surface properties (Zhang et al., 2016). The AFeNPs can target the mild acidity environment of the tumor and kill the cancer cell by inducing a Fenton reaction overproducing H_2_O_2_ in the tumor: the ferrous ion released by the AFeNPs in the tumor, reacting with H_2_O_2_ leading to hydroxyl radical generation. The endogenous hydroxyl radicals generated by AFeNPs enabled to kill cancer cells specifically (Tang et al., 2021).

**FIGURE 2:**
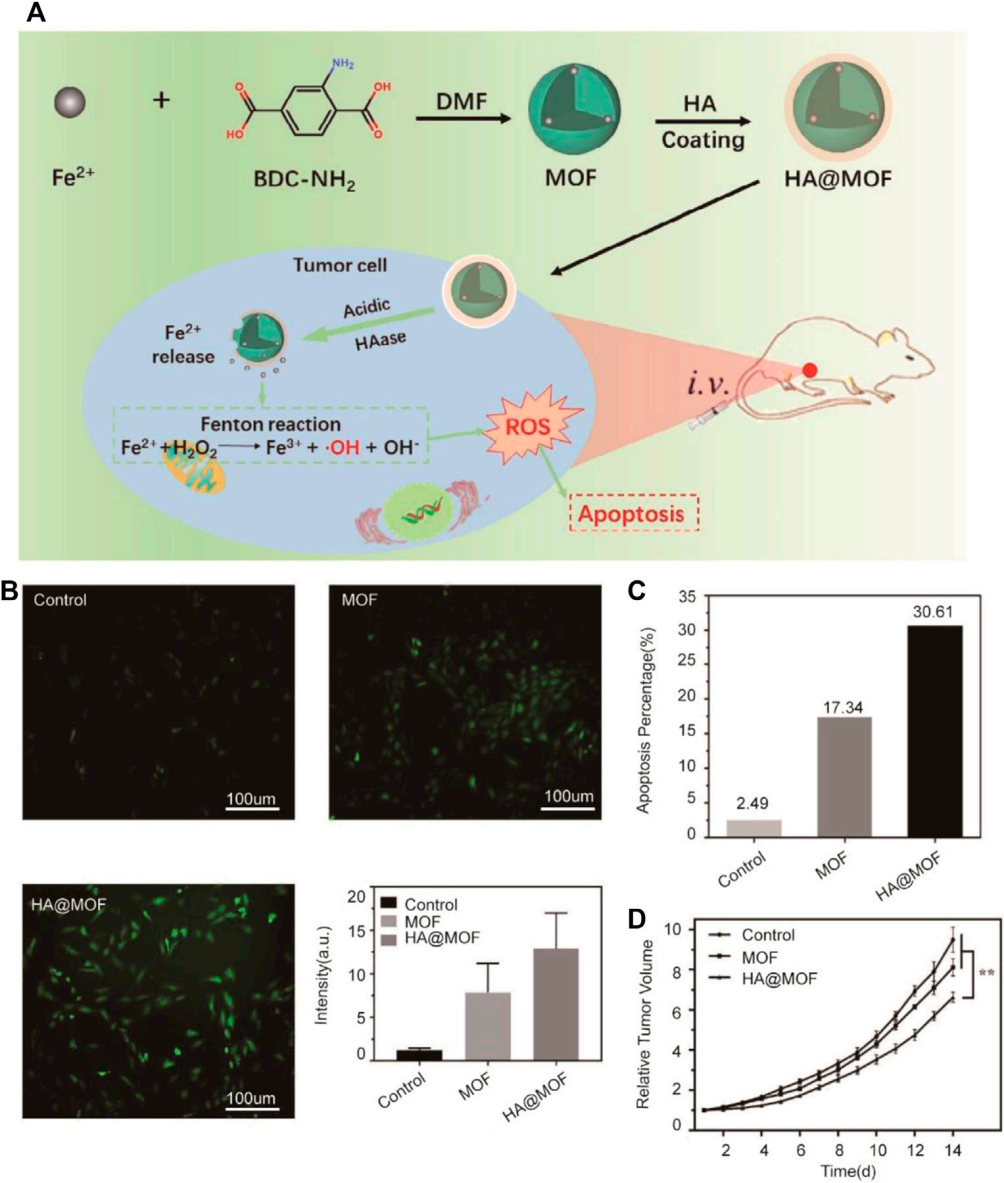
**(A)** Illustration of the synthesis process and mechanism of nanoparticles for tumor therapy. BDC-NH_2_: 2-aminoterephthalic acid; DMF: *N*, *N*-dimethylformamide; MOF: metal-organic framework; HA: hyaluronic acid; **(B)** Graphs show ROS levels of 4T1 cells after treated with the MOF and HA@MOF for 48 h; untreated 4T1 cells were set as control. A quantitative flow cytometry result was also demonstrated. Scale bar = 100 μm; **(C)** Quantitative result of 4T1 cell apoptosis after differently treated; **(D)** Plasma Fe^2+^ concentrations of SD rats injected with MOF and HA@MOF within 12 h (Adapted from Ref. 58 with permission. Copyright © 2020 Journal of Materials Chemistry B).

Ji et al*.* developed the special metal-organic framework (MOF) consisting of FeAc and BDC-NH_2_. MOF was coated with HA to enhance its biostability. With Fe^2+^ delivered into breast cancer cells, the Fenton reaction can be triggered and excessive ROS-inducing cell apoptosis is produced (Xu et al., 2020) (Figure 2). The nanomedicine with excellent stability and pH-responsive ability can target tumors due to its acidic microenvironment of cancer and release Fe^2+^ to tumor cells. The Fenton reaction was induced by excessive Fe^2+^ to inhibit tumor growth due to ferroptosis. Fe^2+^-based MOF proved better biocompatibility and inhibitory effects in *in vivo* experiments.

**FIGURE 3:**
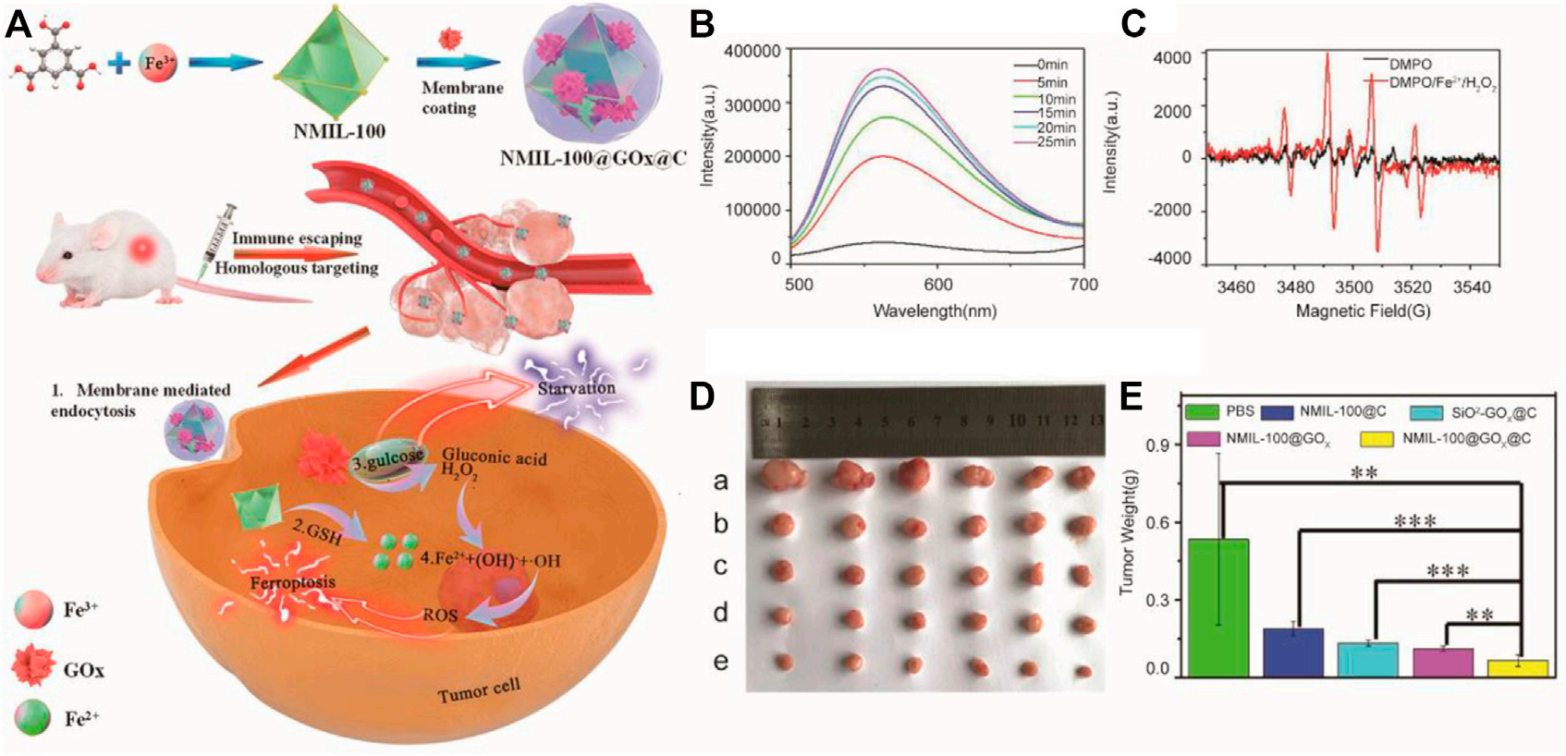
**(A)** Illustration of the synthesis process and mechanism of nanoparticles for tumor therapy; **(B)** Fluorescence intensity of ER-H_2_O_2_ in the NMIL-100@GOx@C solution at different time after addition of glucose; **(C)** ESR analysis of •OH production using DMPO as the spin trapping agent; **(D)** Picture of tumors dissected on the 14th day after different treatments (a: PBS, b: NMIL-100@C, c: SiO_2_−GOx@C, d: NMIL-100@GOx, e: NMIL-100@GOx@C); **(E)** Average tumor weights in different treatment groups.(Adapted from Ref. 59 with permission. Copyright © 2020 ACS Nano) GOx: glucose oxidase.

Furthermore, increasing the concentration of Fe^2+^/Fe^3+^ and H_2_O_2_ at the same time is a more effective strategy to accelerate the Fenton reaction. Li and Tang et al*.* synthesized a Fe^3+^ metal-organic framework (MOF) based on decorative glucose oxidase (GOx), which was coated with the cancer cell membrane as a cascade nanoreactor for synergistic ferroptosis-starvation anticancer therapy (Wan et al., 2020) (Figure 3). Specifically, NMIL-100, a kind of iron-based MOF, was used as the source of iron to induce ferroptosis and a carrier to load GOx. Furthermore, NMIL-100@GOx was coated by the tumor cell membrane to obtain a nanoreactor. In tumor cells, glucose can be catalyzed by GOx to produce excessive H_2_O_2_ for cancer treatment based on ferroptosis, and the glucose reduction caused by GOx, as starvation therapy for tumors, can have a synergistic effect to further inhibit tumors. In mechanism, a large amount of GSH reduced Fe^3+^ from the nanoreactor to cause the collapse of the MOF structure and release Fe^2+^ at the tumor site. Then, H_2_O_2_ generated by the oxidation of glucose catalyzed by GOx reacted with Fe^2+^ to produce hydroxyl radicals to promote ferroptosis-inducing cancer treatment. The nanoreactor loading necessary reactants for the Fenton reaction exhibited a better ability of tumor suppression *in vivo*.

### 2.2 Inducing Ferroptosis by Suppressing the Activity of GPX4

GPX4 (Glutathione peroxidase 4) is the key regulator in ferroptosis. Its inhibitor can promote ferroptosis directly. At the same time, the regulation of GSH, as a substrate of GPX4, is another important approach in ferroptosis (Wu et al., 2021).

#### 2.2.1 Suppressing Function of System Xc^-^

**FIGURE 4:**
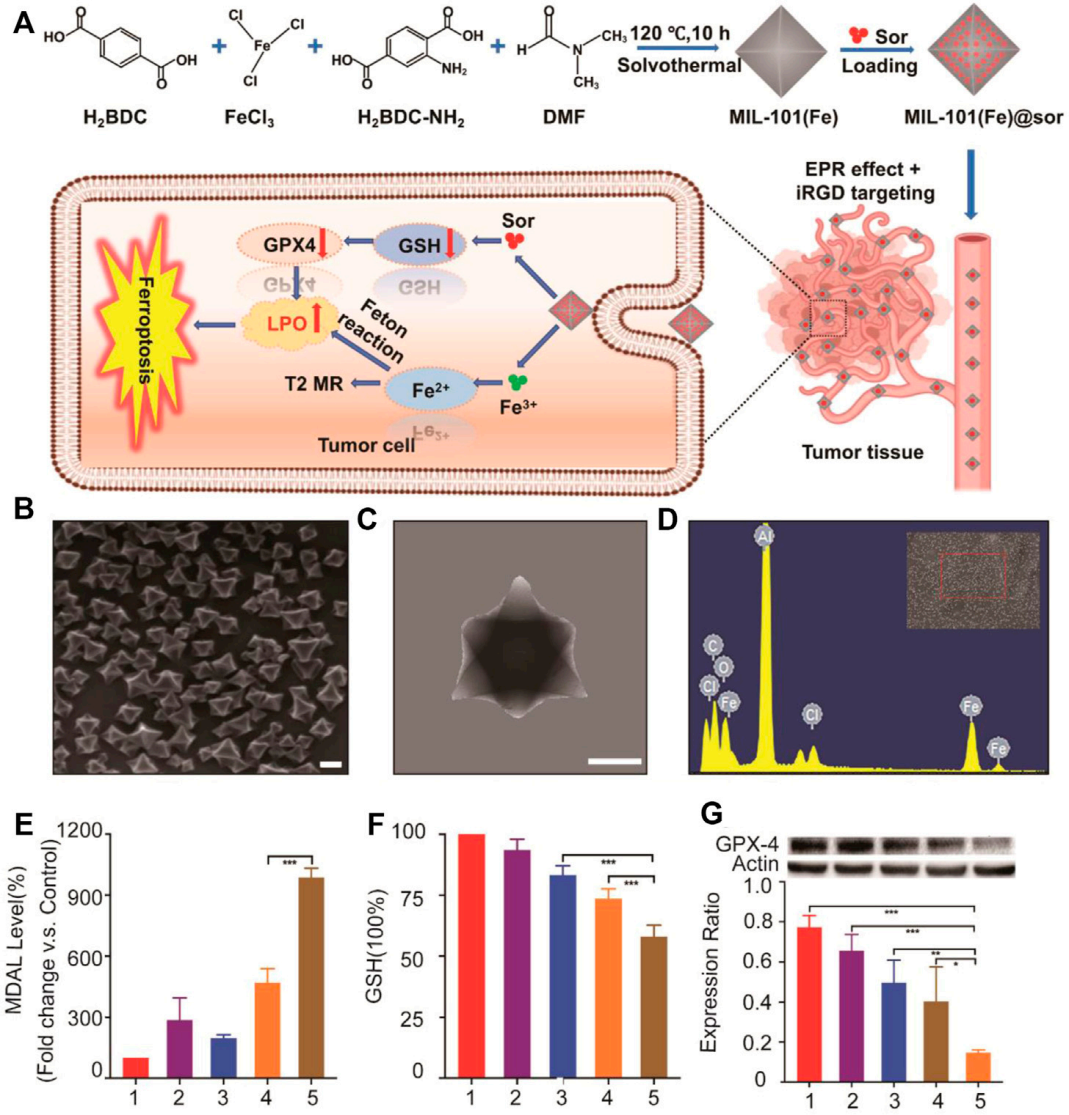
**(A)** Illustration of the synthesis process and mechanism of nanoparticles for liver cancer therapy; **(B)** SEM and **(C)** TEM images of MIL-101(Fe) NPs (scale bar: 100 nm); **(D)** EDS spectrum of MIL-101(Fe) NPs; **(E,F)** MDA and GSH levels in HepG2 cells after different treatments; **(G)** WB analysis of the GPX4 expression in HepG2 cells after different treatments. 1–5: control, MIL-101(Fe), sorafenib, MIL-101(Fe)@sor, and MIL-101(Fe)@sor + iRGD groups, respectively. *p < 0.05, **p < 0.01, ***p < 0.001.(Adapted from Ref. 61 with permission. Copyright © 2021 International Journal of Nanomedicine) Sor: sorafenib.

Besides focusing on the Fenton reaction, a research by Xu et al*.* tried to increase concentration of iron and suppress the function of system Xc-simultaneously (Liu et al., 2021b) (Figure 4). In this study, sorafenib (sor)-loaded Fe-metal organic framework nanoparticles were conjugated with the iRGD peptide to form the multifunctional nanocomposite, MIL-101(Fe)@sor. This nanoparticle can not only effectively induce tumor ferroptosis but also enhance the nanodrug tumor-targeting and penetration abilities. Liver tumors could be eliminated after MIL-101(Fe)@sor nanoparticle treatment, with a significantly prolonged survival period of tumor xenograft mice.

#### 2.2.2 Depletion of GSH

**FIGURE 5:**
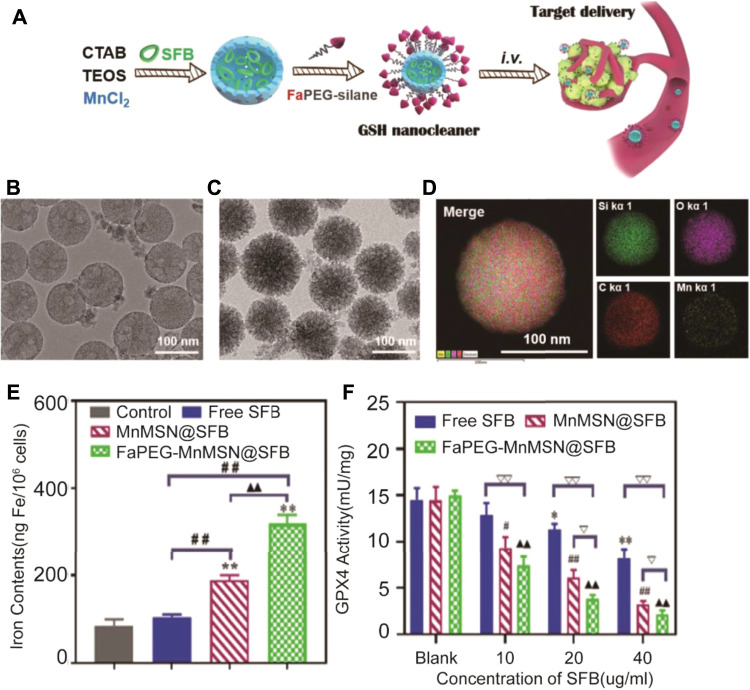
Illustration of the synthesis process and mechanism of nanoparticles for tumor therapy; **(B)** TEM images of MnMSN and **(C)** FaPEG-MnMSN; **(D)** Element mappings of FaPEG-MnMSN; **(E)** Iron contents in HepG2 cells after treated with free SFB, MnMSN@SFB and FaPEG-MnMSN@SFB at the concentration of 20 μg/ml; **(F)** **p < 0.01 *vs.* Blank group. ^##^ *p* < 0.01 *vs.* cells treated with FaPEG-MnMSN@SFB. ▲▲*p* < 0.01 *vs.* cells treated with MnMSN@SFB. GPx4 activity of HepG2 cells after treated with free SFB, MnMSN@SFB and FaPEG-MnMSN@SFB for different concentrations. (Adapted from Ref. 62 with permission. Copyright © 2020 Theranostics) CTAB: cetyltrimethylammonium bromide; TEOS: tetraethyl orthosilicate; SFB: sorafenib tosylate.

In addition to research studies focusing on the block transport system Xc^−^, Xu et al*.* developed nanoparticles (FaPEG-MnMSN@SFB) inducing ferroptosis by both suppressing function of system Xc^−^ and depletion of GSH (Tang et al., 2020) (Figure 5). MnMSN@SFB was synthesized with MnMSN and sorafenib (SFB) by the optimized one-pot Stober’s method. The surface of MnMSN was modified with the FaPEG chain to achieve better stability in circulation and delivery processes. On the one hand, the manganese–oxygen bond (‒Mn‒O‒) in MnMSN leaded to consumption of GSH in the cell. On the other hand, SFB, as an inhibitor of the Xc^−^ transport system could inhibit the synthesis of GSH. Therefore, the nanoparticles exhibited efficient antitumor activity under dual roles. Moreover, apoptosis could be induced by disruption of redox balance producing the synergy effect with ROS-dependent ferroptosis to tumor cells.

## 3 Emerging Nanomedicine Based on Ferroptosis and Conventional or Emerging Tumor Therapy

Currently, although emerging tumor treatment methods have made some progress, the effect of them is still difficult to be satisfied in the clinical treatment (Lai et al., 2021; Mo et al., 2021; Ou et al., 2021; Wang and Bai, 2021). Ferroptosis has the potential ability to make the synergy effect with conventional or emerging tumor therapy which brings more spark in the treatment of different tumors (Xue et al., 2020).

### 3.1 Combination of Ferroptosis With Chemotherapy

**FIGURE 6:**
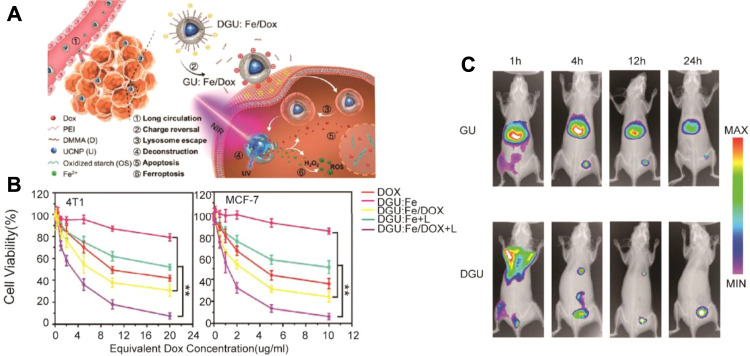
Schematic illustration of nanolongan; **(B)** CCK-8 cytotoxicity analysis of 4T1 and MCF-7 cells treated with different formulations after 24 h of incubation; **(C)** *In vivo* imaging of biodistribution of GU and DGU in the tumor-bearing mouse model. (Adapted from Ref. 70 with permission. Copyright © 2019 ACS Nano) UCNP: upconversion nanoparticle; Dox: doxorubicin; PEI: polyethylenimine; DMMA: 2,3-dimethylmaleic anhydride.

Chemotherapy is a conventional tumor treatment method. Due to lack of specificity for tumor cells, it brings cytotoxicity to any cells. Besides, drug resistance makes its therapeutic effect limited (Ji et al., 2016; Patil et al., 2016). Li et al*.* designed a nanolongan delivery system. The oxidized starch-based gel nanoparticle was coated by the upconversion nanoparticle (UCNP) and doxorubicin (Dox) (query). The carboxyl groups on oxidized starch polymers were coordinated with Fe^3+^ and further decorated with polyethyleneimine (PEI) and 2,3-dimethylmaleic anhydride (DMMA) (Bao et al., 2019) (Figure 6). The negatively charged surface of nanolongan due to DMMA could achieve long circulation after intravenous injection and specially targeted tumor delivery *via* the EPR effect. After the nanolongan reached the tumor site with a weak acidic microenvironment, the negatively charged surface of nanolongan could be converted with a positive charge due to exfoliation of DMMA, which facilitated its internalization of cancer cells and escape of lysosomes due to the proton-sponge effect. With further near-infrared (NIR) light irradiation, Fe^3+^ can be converted to Fe^2+^ by UCNP in the cancer cell, which further promoted the Fenton reaction to induce ferroptosis-generating synergy effect with apoptosis due to Dox.

### 3.2 Combination of Ferroptosis With Immunotherapy

**FIGURE 7:**
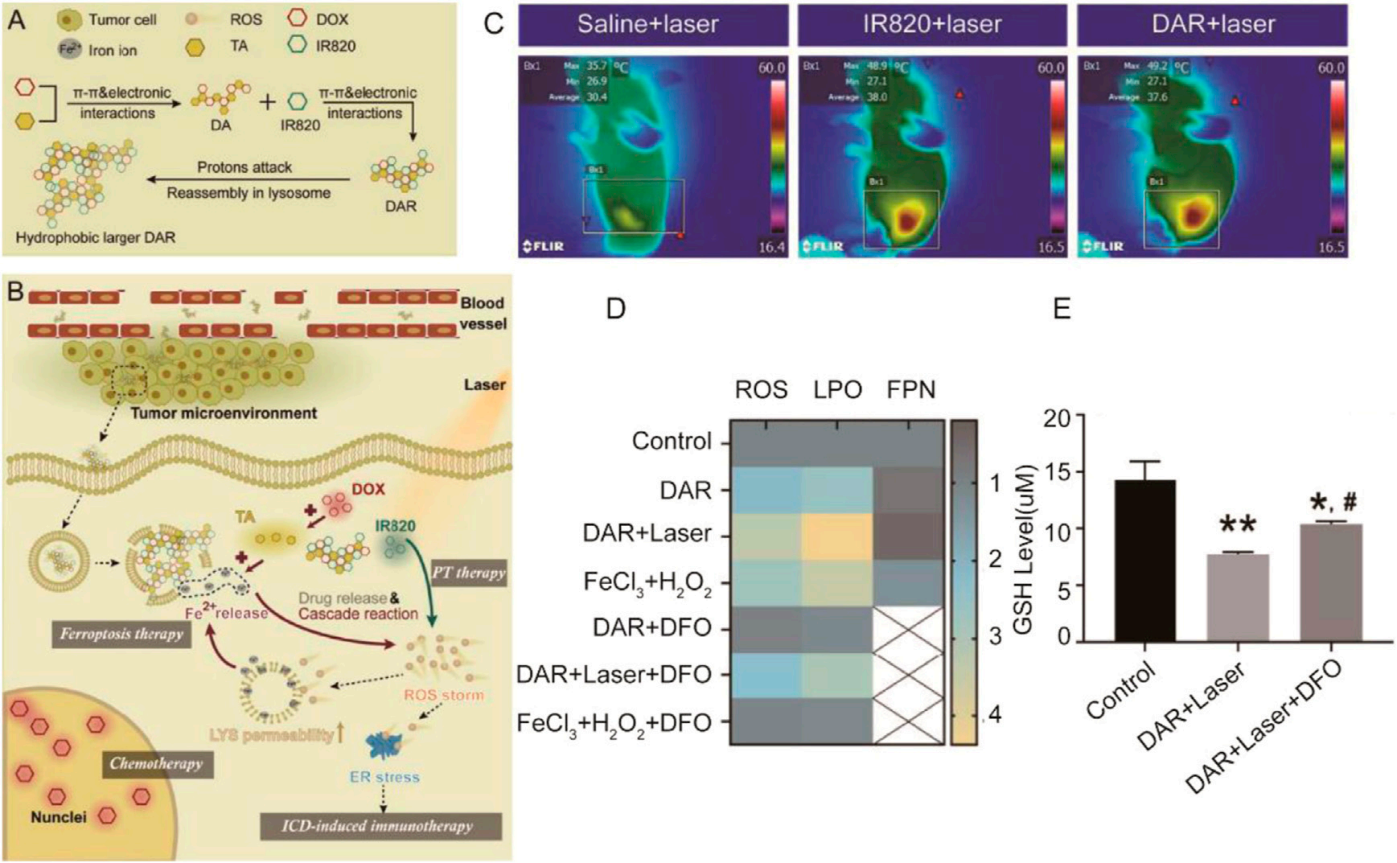
**(A)** Assembly and reassembly processes of DAR; **(B)** The tumor delivery, tumor cell uptake and intracellular transition, lysosome escape, and laser-promoted drug release processes of DAR. LYS and ER were abbreviations of lysosome and endoplasmic reticulum, respectively; **(C)** Infrared thermographic images of mice injected with saline; IR820 and DAR were tested at 5 min after laser irradiation; **(D)** Relative concentration of ROS, LPO, and FPN of MCF7 cultured in different drugs or agents (*n* = 3); **(E)** GSH levels of MCF7 cells treated with different formulations (*n* = 3). *p < 0.05 vs. control; **p < 0.01 vs. control; ^#^ *p* < 0.05 vs. DAR + laser. (Adapted from Ref. 77 with permission. Copyright © 2021 Journal of Controlled release).

Tumor immunotherapy is an emerging tumor therapy method by regulating the tumor microenvironment to enhance the antitumor immunity. It provides a new idea for tumor treatment, but its effectiveness and safety need to be further studied (Fang et al., 2021; George et al., 2021). Studies have shown that immunotherapy-activated T cells can enhance ferroptosis-specific lipid peroxidation in tumor cells, and in turn, increased ferroptosis contributes to the antitumor efficacy of immunotherapy. Therefore, combination of ferroptosis with immunotherapy has been a new strategy for design of nanomedicine (Jiang et al., 2020; Yang et al., 2021b; Liang et al., 2021). In Yao et al.’s research, a nanoactivator (DAR) was constituted by doxorubicin (DOX) (query), tannic-acid (TA), and IR820. During the process of synthesis of DAR, DOX, as a chemotherapeutic drug, interacts with TA, as a disproportionation activator, to form intermediate products (DA) at the first step. Then, DA made the interaction with IR820, as a photothermal therapy agent, to form DAR finally through π–π and electronic interactions (Xiong et al., 2021) (Figure 7). After the nanoactivator is injected into the cell through endocytosis, with the IR820 responding to the laser, DAR can be reassembled quickly to realize the escape of lysosomes and promote the rapid release of endogenous Fe^2+^ due to the acidic microenvironment of lysosomes. The released Fe^2+^ accelerated the Fenton reaction to promote ferroptosis and the accumulation of ROS. Excessive ROS further induced the immune response induced by DOX to kill tumor cells. In contrast, immunotherapy would activate the infiltration of CD8^+^ T cells in tumor cells, and ferroptosis through IFN-γ–related pathways was promoted. The nanoactivator reflected a very significant tumor killing ability through the synergistic effect of immunotherapy and ferroptosis.

A biomimetic magnetosome was prepared by Xie et al*.*, composed of Fe_3_O_4_ magnetic nanoclusters (NCs), pre-engineered leukocyte membranes, TGF-β inhibitor (Ti), and PD-1 antibody (Pa) (Zhang et al., 2019). Specially, the NC was coated by leukocyte membranes pre-engineered with azide (N_3_) with modified Ti and Pa to form Pa-M/Ti-NCs. After intravenous injection, the Fe_3_O_4_ magnetic nanoclusters break through the biological barrier and are accumulated to the tumor. The Pa and Ti on the surface of Pa-M/Ti-NCs would increase the amounts of CD4^+^ T/Treg cells, CD8^+^ T/Treg cells, and rate of producing M1/M2 to induce immunogenicity of macrophages. In addition, M1 polarization due to immune response increased the amount of H_2_O_2_. With the release of ions, it promoted the Fenton reaction with H_2_O_2_ and induced ferroptosis of tumor cells.

### 3.3 Combination of Ferroptosis With Gene Therapy

**FIGURE 8:**
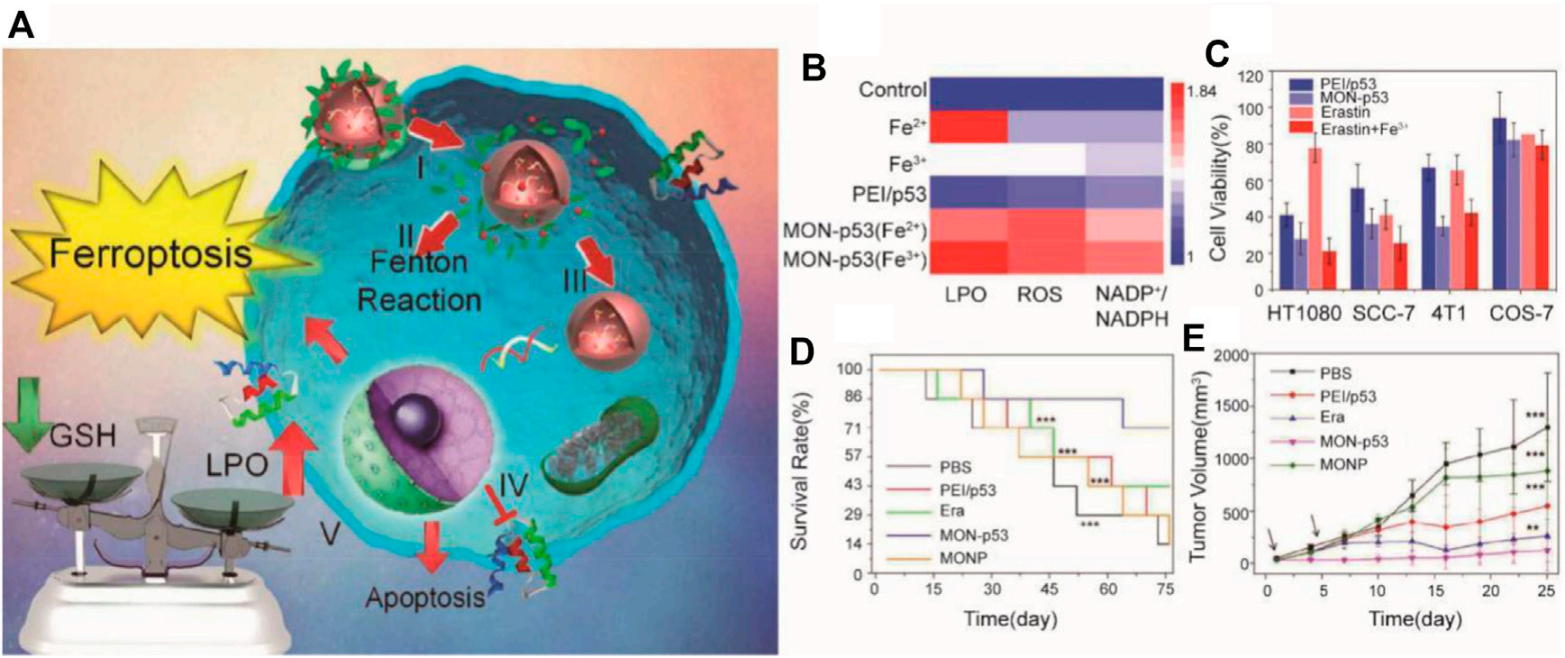
**(A)** Illustration of the synthesis process and mechanism of nanoparticles for tumor therapy; **(B)** LPO, ROS, and 5 NADP+/NADPH content of HT1080 cells treated with ferric chloride (Fe(III), 10 μM), 6 (Fe(II), 25 μM), PEI/p53, Fe(II)-MON-p53, and Fe(III)-MON-p53; **(C)** Viability of 7 HT1080 cells, SCC-7 cells, 4T1 cells, and COS7 cells after treatment with PEI/p53, 8 MON-p53, erastin, and erastin + Fe(III); **(D)** Survival curves of mice receiving injections of Era at a dose of 5 mg/kg, 3 MONP at a dose of 5 mg/kg, and a DNA dose of 0.375 mg/kg (*n* = 7 for all groups) in 4 HT1080 tumor-bearing mice; **(E)** HT1080 tumor volume curves of mice at the first 25 days (Adapted from Ref. 81 with permission. Copyright © 2017 Nano Letters) MON: metal organic network.

Tumor gene therapy mainly targets tumor suppressor genes, immunostimulatory genes, and anti-angiogenic factors. Among them, adenovirus carrying p53 has the fastest development in clinical research. However, the specific mechanism, long-term efficacy, and adverse effects of gene therapy are still unclear (Chen et al., 2001; Zhang et al., 2011). In Zhang et al.’s research, breast cancer cells were eradicated by the metal organic network encapsulated with the p53 plasmid (MON-p53) *via* the ferroptosis/apoptosis hybrid pathway (Zheng et al., 2017) (Figure 8). As we know, p53 is a tumor suppressor, so a “bystander effect” was mediated by MON-p53 to further sensitize cancer cells toward the MON-p53 inducing ferroptosis and gene therapy. MON-p53 treatment has two effects, which both suppressed the tumor growth and prolonged the lifespan of tumor-bearing mice; it was found in an anticancer experiment.

### 3.4 Combination of Ferroptosis With Ultrasound Therapy

**FIGURE 9:**
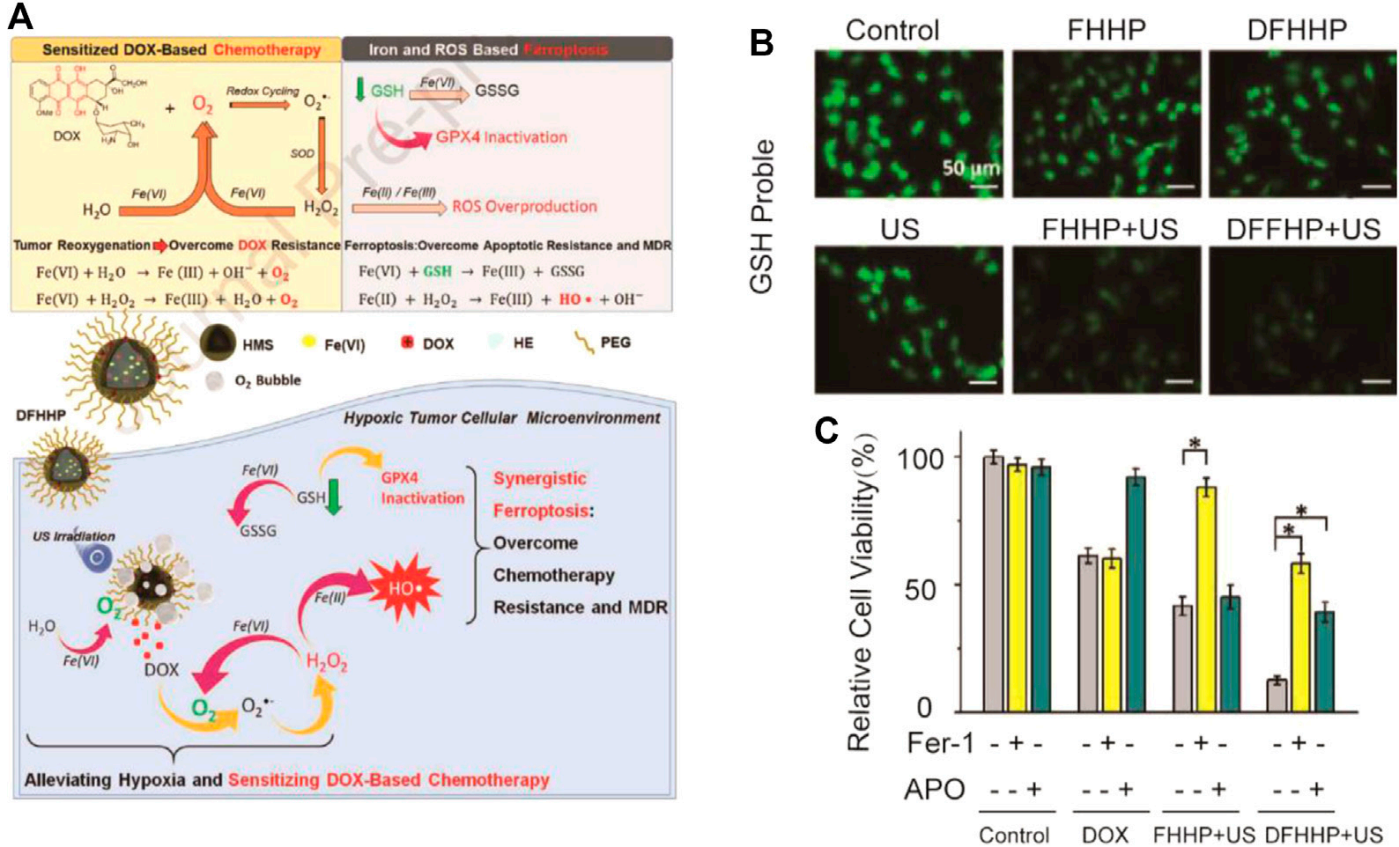
**(A)** Illustration of the synthesis process and mechanism of nanoparticles for tumor therapy; **(B)** Fluorescence images of Saos-2 cells after different treatments. The cellular GSH level was indicated by Thiol-Tracker Violet. Cells in fresh culture media were used as the control group; **(C)** *In vitro* cytotoxicity of DOX, FHHP + US, and DFHHP + US against Saos-2 cells after the pretreatment with the ferroptosis inhibitor ferrostatin-1 (Fer-1) or apoptosis inhibitor Ac-DEVD-CHO (Apo). (Adapted from Ref. 84 with permission. Copyright © 2020 Biomaterials).

Ultrasound can pass through the body and focus on deep tumor tissues. High-intensity ultrasound can kill tumors by high temperature. The combination of ultrasound therapy and nanotechnology can be a switch for nanomedicine to achieve precise drug release (Wang et al., 2017; Zhu et al., 2018). A nanomedicine with low-dose ultrasound responding was designed in Hao et al.’s research through combining DOX with the nanoplatform, subsequently incorporating *n*-heneicosane and polyethylene glycol chains (Fu et al., 2021) (Figure 9). Due to solid–liquid phase change, *n*-heneicosane could respond to mild high temperatures caused by ultrasound generating ultrasound-responsive cargo. Nanoparticles extravasate into the osteosarcoma tumor through the enhanced permeability and retention (EPR) effect of the tumor. On the one hand, the sensitivity of the apoptosis mediated by DOX of tumors can be caused by ultrasound-responsive DFHHP nanomedicine (DOX-Fe(VI)@HMS-HE-PEG, abbreviated as DFHHP) *via* alleviating the hypoxic tumor microenvironment by tumor reoxygenation. On the other hand, exogenous iron and systemic GPX4 could cause inactivation-efficient ferroptotic cell death. The US-activatable nanomedicine overcame chemoresistance and suppressed tumor growths by inducing collaborative apoptosis and ferroptosis of hypoxic osteosarcoma *in vitro* and *in vivo*.

### 3.5 Combination of Ferroptosis With Photodynamic Therapy

Photodynamic therapy uses lasers of specific wavelengths to activate photosensitizers selectively retained in tumor tissues to selectively kill tumor cells (Patrice, 1991; Blasi et al., 2018). Zhao et al*.* provided a new treatment strategy for triple-negative breast cancer (TNBC) with azobenzene combretastatin A4 (Azo-CA4) (Zhu et al., 2020). Near-infrared light was converted to UV light by upconverting nanocarriers (UCNPs) to activate Azo-CA4, which being physically encapsulated in the lipid (LP) bilayer and was loaded in UCNPs. After irradiation, the viability of TNBC cells was significantly reduced by UCNP@LP (Azo-CA4) nanocarriers through both apoptosis and ferroptosis. On the one hand, microtubule breakdown and cell cycle arrest at the G2/M phase was induced by photoisomerization of Azo-CA4. On the other hand, the UV light–induced reduction of Fe^3+^ to Fe^2+^ caused ferroptosis, which facilitated the peroxidation of lipids. The tumor growth of xenograft mice was significantly suppressed by UCNP@LP (Azo-CA4).

A novel theranostic nanoplatform was developed in Zheng et al.’s research for tumor chemodynamic–photothermal therapy and 3D imaging diagnosis (Hu et al., 2020). By the synthesis of Prussian blue cubes (PB) and *in situ* reduction of iron platinum nanoparticles (FePt-NPs) in a facile way and coating with targeting ligands (hyaluronic acid) and NH_2_-PEG, the nanoplatform was successfully fabricated. The nanoplatform has two functions: it decomposes endogenous H_2_O_2_ into ROS, which is highly cytotoxic to tumor cells and produces a multifunctional theranostic agent for multimodal imaging and therapies. Moreover, the specific tumor-targeting ability to the nanoplatform was given by HA, with excellent biocompatibility and biological degradability, due to binding with CD44 and CD168. The tumor growth of xenograft mice was effectively suppressed by intravenous injection of the nanoplatform, indicating that tumor growth efficaciously inhibited the nanoplatform, with relatively high biosafety.

## 4 Conclusion and Prospective

Cancer is a public health problem worldwide, whose incidence of tumor increases year by year. At present, traditional treatment of tumor is usually combined with chemotherapy, radiotherapy, and other methods. However, there are large side effects, easy to produce drug resistance, and other problems during the process of treatment. Therefore, in recent years, gene therapy, immunotherapy, and other emerging treatment methods have been widely studied. However, the biosecurity, specific mechanisms, and long-term therapeutic effects of these emerging treatments need to be further studied. Ferroptosis is a new form of regulated cell death that is defined in 2012. In recent years, a large amount of research has focused on the mechanisms of ferroptosis. Based on these mechanisms, especially for the Fenton reaction and suppressing activity of GPX4, ferroptosis has provided a new idea for cancer therapy. However, tumor cells can adapt to the microenvironment through metabolic change; some small molecules, such as the ferroptosis inducers, that merely increase the concentration of ROS in tumor cells or inhibit the activity of GPX4 cannot achieve strong and lasting antitumor effects. In a way, the small molecules lack tumor specificity, and they are easier to be eliminated during blood circulation.

With the development of nanomaterial technology, ferroptosis-inducing nanomedicines have attracted more attention. Therefore, it is timely to outline the latest advances in ferroptosis-inducing nanomedicines. The combination inducing ferroptosis with nanotechnology enhances the stability, biosecurity, targeting, and controlled release of drugs in the body. Specifically, the advantages of ferroptosis-inducing nanomedicines include three aspects. First, the nanomaterials with a regulated size can facilitate passive targeting to the tumor microenvironment (EPR effect). By modifying the nanoparticles with specific antibodies against the tumor surface, the active targeting of nanomedicine to tumors can also be achieved. Second, the large hydrodynamic size of nanomaterials can reduce the renal clearance and prolong the half-life in the blood. Moreover, ferroptosis-inducing nanomedicines combined with other therapies, such as chemotherapy, gene therapy, ultrasound therapy, and photodynamic therapy, can achieve more effective treatments with the synergy effect. To a certain extent, ROS produced by ferroptosis can regulate the tumor microenvironment and further induce cell apoptosis, enhance ability to kill tumor cells of nanomedicine, or even reverse tumor resistance. In addition, a large number of studies have also shown that autophagy is indeed induced by the ferroptosis inducer, and autophagy can enhance ferroptosis in cancer cells by degradation of ferritin (Hou et al., 2016; Torii et al., 2016; Park and Chung, 2019). Chen et al*.* constructed trehalose-loaded mSiO_2_@MnO_x_-mPEG (TreMMM) nanoparticles, its combination of GSH consumption-induced ferroptosis and trehalose-induced autophagy by nanomedicine design, providing a new strategy for the design of ferroptosis-inducing nanomedicines (Yang et al., 2021c). Besides, several other key molecules in the NRF_2_ pathway or RAS/MAPK pathway are related to ferroptosis, which provides an opportunity to find out various targets to design nanomedicines (Yagoda et al., 2007; Roh et al., 2017; Wang et al., 2021d).

Although ferroptosis-inducing nanomedicines have been developed rapidly, the potential clinical application remains to be further investigated. On the one hand, most of the current research focuses on cells or animal models; there might be a difference between the human body and the models used in most research, such as cells and animals. It is questionable that whether ferroptosis-inducing nanomedicines have effective treatments in the human body. On the other hand, whether ferroptosis-inducing nanomedicines have potential toxic and side effects that should be further investigated. Moreover, existing research of ferroptosis-inducing nanomedicines is mainly a benefit to breast cancer and liver cancer. This may be due to the high incidence of these tumors, which is suitable as the model for tumor therapy. Meanwhile, different tumors have different sensitivities to the ferroptosis-inducing nanomedicines because of their adaptability to ROS. Therefore, it is necessary to further investigate the mechanisms of tumor resistance. Importantly, the research on the mechanism of gene therapy and immunotherapy, as emerging treatment methods, is not thorough enough. Whether the combination with inducing ferroptosis will cause other side effects remains to be further studied. In summary, although ferroptosis-inducing nanomedicines have achieved the positive antitumor effect on the research, the treatment of tumors based on ferroptosis is in the initial stage. In the future, many issues need to be clarified in combination of biochemistry, oncology, and material technology to design the effective and safe cancer therapy strategies based on ferroptosis.
